# Impacts of the COVID‐19 pandemic on livelihoods and wild meat use in communities surrounding the Dja Faunal Reserve, South‐East Cameroon

**DOI:** 10.1111/aje.12995

**Published:** 2022-03-25

**Authors:** Cédric Thibaut Kamogne Tagne, Stephanie Brittain, Francesca Booker, Dan Challender, Neil Maddison, Eleanor Jane Milner‐Gulland, Mama Mouamfon, Dilys Roe, Lauren Coad

**Affiliations:** ^1^ Fondation Camerounaise de la Terre Vivante (FCTV) Yaoundé Cameroon; ^2^ Department of Zoology Interdisciplinary Centre for Conservation (ICCS) University of Oxford Oxford UK; ^3^ International Institute for Environment and Development (IIED) London UK; ^4^ The Conservation Foundation (TCF) London UK; ^5^ CIFOR Jalan CIFOR Situ GedeBogor BaratBogorIndonesia

**Keywords:** bushmeat, Cameroon, COVID‐19, livelihoods

## Abstract

The COVID‐19 outbreak has had considerable negative impacts on the livelihoods and living conditions of communities around the world. Although the source of COVID‐19 is still unknown, a widely spread hypothesis is that the virus could be of animal origin. Wild meat is used by rural communities as a source of income and food, and it has been hypothesised that the pandemic might alter their perceptions and use of wild meat. McNamara et al. (2020) developed a causal model hypothesising how the impacts of the pandemic could lead to a change in local incentives for wild meat hunting in sub‐Saharan African countries. From February 27 to March 19, 2021, we carried out a survey around the Dja Faunal Reserve, Southeast Cameroon, to test McNamara et al.’s model in practice, using semi‐structured questionnaires to investigate the impacts of the COVID‐19 outbreak on wild meat hunting and consumption. Our results generally agree with the causal pathways suggested by McNamara et al. However, our study highlights additional impact pathways not identified in the model. We provide revisions to McNamara's model to incorporate these pathways and inform strategies to mitigate the impacts of the pandemic.

## Résumé

L'épidémie de COVID‐19 a eu des répercussions négatives considérables sur les moyens de subsistance et les conditions de vie des communautés du monde entier. Bien que l'origine de la COVID‐19 soit encore inconnue, une hypothèse largement répandue est que le virus pourrait être d'origine animale. La viande de gibier est utilisée par les communautés rurales comme source de revenus et de nourriture et une hypothèse avance que la pandémie pourrait modifier la perception et l’utilisation de ces dernières à l’égard de cette même viande McNamara et al. (2020) ont élaboré un modèle de causalité en émettant l'hypothèse que les répercussions de la pandémie pourraient entraîner une modification des incitations liées à la chasse de gibier au niveau local dans les pays d'Afrique subsaharienne. Entre le 27 février et le 19 mars 2021, nous avons effectué une étude aux alentours de la réserve de faune du Dja, au sud‐est du Cameroun, afin de tester le modèle de McNamara et al. dans la pratique, en nous appuyant sur des questionnaires semi‐structurés afin d’étudier les répercussions de l'épidémie de COVID‐19 sur la chasse de gibier et la consommation de viande de gibier. Nos résultats correspondent globalement au modèle causal suggéré par McNamara et al. Cependant, notre étude met en évidence d'autres schémas de répercussions non identifiés dans ce modèle. Nous souhaitons apporter des modifications au modèle de McNamara afin d'intégrer ces schémas, ainsi que des stratégies visant à atténuer les impacts de la pandémie.

## 1 INTRODUCTION

The COVID‐19 pandemic has shone a spotlight on the sale and consumption of wild meat (e.g. Booth et al., 2021; Borzée et al., 2020; Roe & Lee, 2021; Rzymski et al., 2021). Whilst the source of the COVID‐19 outbreak is still unknown, one widely‐cited hypothesis was that the virus could have originated in a bat species, and that transmission to humans may have occurred through the sale of wild meat at ‘wet markets’ in Wuhan, China (Zhou et al., 2020). This led to calls to more stringently regulate, or even stop all commercial trade in wildlife for human consumption and close wildlife markets (e.g. Aguirre et al., 2020; Borzée et al., 2020; WCS, 2020). Wild meat is used by rural communities around the world as a regular source of food and income (Coad et al., 2018) and a ban on sales of wild meat could, therefore, have significant impacts on both food security and local livelihoods (Roe & Lee, 2021). However, regardless of the potential impact of trade bans, the economic and social shocks created by the COVID‐19 pandemic are likely to impact local consumption and trade of wild meat by rural communities.

**FIGURE 1:**
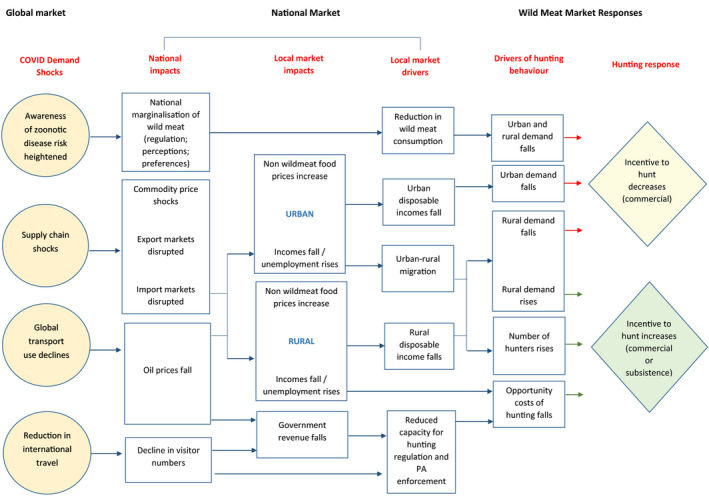
Causal model describing key linkages between global COVID‐linked shocks and wild meat market dynamics. Reproduced from McNamara et al., 2020, with permission

McNamara et al. (2020) developed a causal model that tracks the likely implications for the wild meat trade of the systemic crisis triggered by COVID‐19 in sub‐Saharan Africa (Figure 1). They hypothesise that the negative impacts of the global pandemic on food supply chains, and national and local economies and employment levels, could result in changes in incentives for wild meat hunting in sub‐Saharan African countries. In urban areas, where wild meat is often consumed as a luxury good, reduced household incomes may lead to a reduction in its consumption, with a subsequent knock‐on impact on the trade from rural to urban areas. Reduced urban incomes may also result in migration of individuals or entire households from urban areas back to natal villages, where living costs are lower. This may have the opposite effect of increasing demand for wild meat in rural areas, and the number of individuals hunting for subsistence purposes. Where rural incomes fall, households may fall back on wild meat as a ‘safety net’, increasing their hunting effort to provide food and income (McNamara et al., 2020). At a national level, reductions in government revenues (such as through oil or tourism) may reduce the funds available for hunting regulation and law enforcement, reducing the opportunity costs of hunting and, therefore, increasing the incentive to hunt. However, on the other hand, increased national awareness of the risk of zoonotic disease transmission from wild animals may result in tougher regulation of wild meat and reduced demand from consumers, reducing the incentive to hunt (McNamara et al., 2020). The overall impact of these different positive and negative economic and regulatory levers on wild meat use, and whether the pandemic increases or decreases use, will depend on which levers are most relevant in a specific area.

This study aims to test McNamara et al.’s model in practice, using semi‐structured questionnaires to investigate how the COVID‐19 outbreak has impacted wild meat hunting and consumption in rural communities surrounding the Dja Faunal Reserve, Southeast Cameroon. In common with all Central African countries, Cameroon has registered few COVID‐19 infections and deaths in comparison to the levels seen in other regions of the world (although testing capacity is also lower); recent estimates put the total number of COVID‐19 cases since January 2020 at over 81,000 with 1,330 deaths ((World Health Organisation, 2021; accessed 20/07/2021), To put this into context, in 2019 there were 4,510 reported malaria deaths in Cameroon (World Health Organisation, 2020). However, global economic shocks, changes in government policies in response to COVID‐19, and national and local perceptions of zoonotic disease risk may all have important livelihood impacts for rural communities already operating at a subsistence level, including their use of wild meat.

To investigate these potential impacts, we carried out a survey of 199 households in 18 villages in the Dja region. We explored the current livelihood activities of respondents, their wild meat consumption patterns, changes in livelihoods and wild meat use during the pandemic, sources of information on COVID‐19 and trust in these sources, perceptions of zoonotic disease risk and levels of support for the idea of banning wild meat markets to prevent future outbreaks. We discuss our results in relation to the causal model suggested by McNamara et al. (2020), providing a real‐world empirical example of the systemic drivers of changes in incentives to hunt wild meat. We also make recommendations on how best to tackle the linkages between public health, wildlife conservation and poverty in a way that recognises the needs and priorities of local communities.

## 2 METHODS

### 2.1 Study site

The Dja Faunal Reserve (from herein ‘the Dja’) is situated between the Eastern and Southern Regions of Cameroon. Surrounded on three sides by the Dja River, the reserve is one of the largest rainforests in Africa and is home to several threatened mammal species (Bruce et al., 2018), including the endangered forest elephant (*Loxodonta cyclotis*) and the critically endangered western lowland gorilla (*Gorilla gorilla gorilla*). The reserve is divided into four administrative sectors (North, East, South and West).

Most communities living around the Dja rely on subsistence farming for food security and livelihoods and use wild meat hunting as an additional source of livelihood support and protein (Bobo et al., 2015). The distinction between subsistence and commercial use of wild meat is often blurred (see Ingram et al., 2021; Ndibalema & Songorwa, 2008), and rural hunters often hunt to both supply the demand for wild meat in urban areas, and for their own subsistence purposes in rural areas. Studies that quantify the role that wild meat hunting plays for both livelihoods and food security around the Dja Reserve are outdated and unlikely to reflect current wild meat use (see Ngnegueu & Fotso, 1996).

**FIGURE 2:**
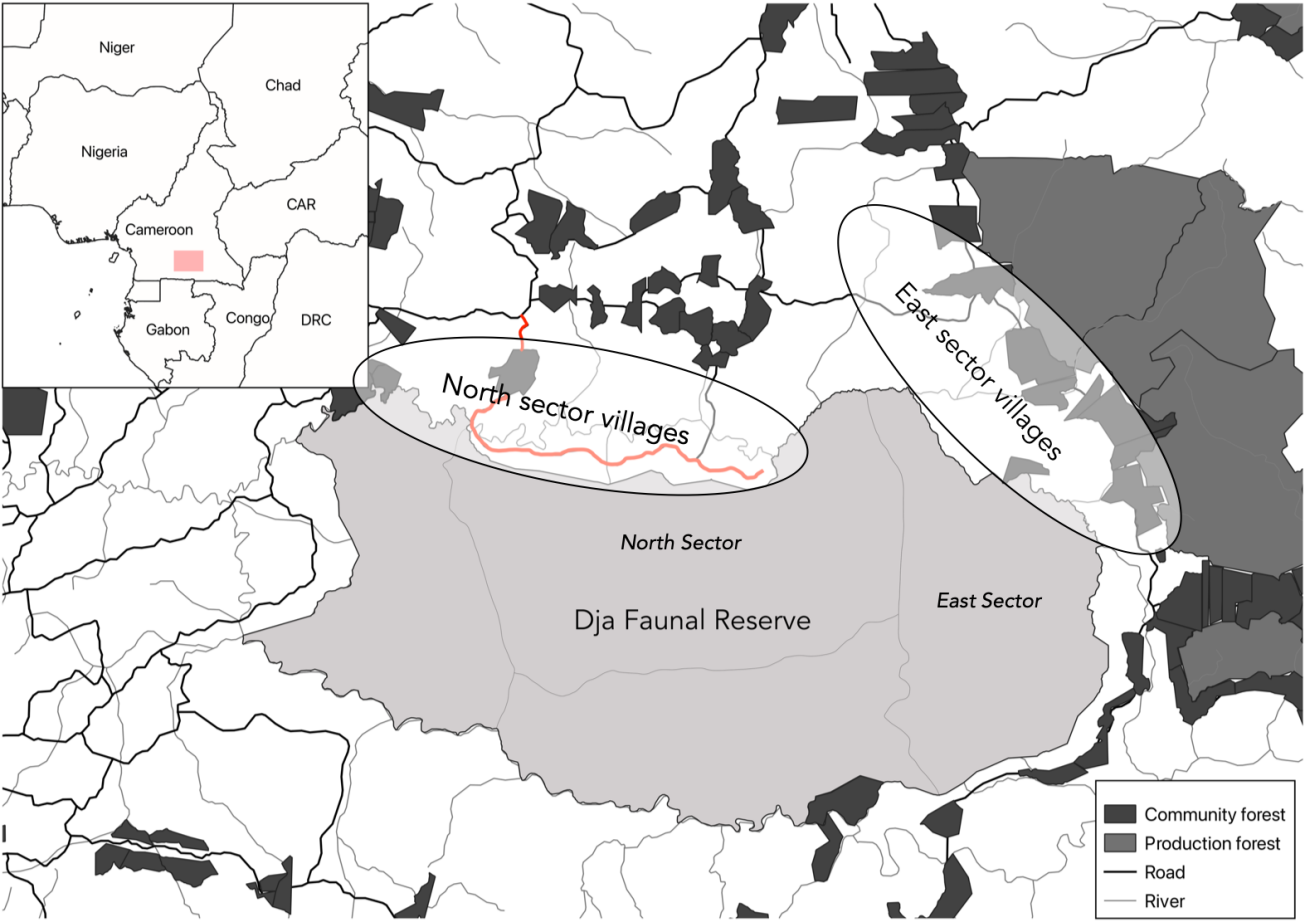
Location of the study sites in the Dja Reserve. Exact locations of villages are not shown to preserve anonymity. The ‘Boucle du Dja’ road is shown here in red, and the four ‘sectors’ (administrative departments of the Dja Reserve), are shown in light grey

We carried out our study in 18 villages around the Dja (Figure 2). Of the 11 study villages in the Northern sector, 10 were found in the ‘Boucle du Dja’, a stretch of road situated in the Northern sector of the reserve. The villages situated along the Boucle are separated from the municipality of Somalomo by the Dja River. During the time of our study, the ‘bac’ (mechanised floating platform) that once transported cars, people and goods across the river, had been broken for over 5 years and people relied on small boats to get across the river. This can be dangerous, especially during the rainy season from November to February, when the river floods and can double in size. Access difficulties make the living conditions of the local communities precarious. Several conservation NGOs work in the Boucle supporting the livelihood activities of the populations through the promotion of sustainable fishing, cocoa and non‐timber forest product (NTFP) production. The predominant ethnic group in the Northern sector study villages is the Badjoué. At the time of the survey the study villages had between 5 and 18 households (mean of 11), and household sizes varied widely, ranging from more than ten to only one inhabitant. The houses are predominantly rectangular and made of clay, and there is no access to electricity or running water in any of the villages. Four primary schools serve 17 villages in the Boucle and secondary education takes place mainly in Somalomo, where there is also the health centre on which these populations rely.

In the Eastern sector of the reserve the seven study villages are situated around the commune of Lomié, a large settlement with access to water and electricity and a good number of facilities, including a large general market, which sells wild meat. Forestry companies house their employees there and several local conservation NGOs are also based there. The presence of logging operations has favoured the development of the region. In the study villages, the dominant ethnic groups are the Zime and the Badjoué. There is also a large Baka population in this part of the Reserve, sometimes grouped into chiefdoms. Access to electricity in the study villages is common, and telephone reception is accessible. Water is generally provided from boreholes. Villages are larger than in the Northern sector, ranging between 15 and 40 households (mean of 25). The architecture of the houses ranges from rectangular houses made of rammed earth, to more sophisticated houses in cement. Some primary schools can be found in the villages although secondary education takes place principally in Lomié and other towns. Medical care is provided mainly in Lomié, but doctors regularly visit the villages. The Eastern study villages are, therefore, close to well‐maintained roads and logging concessions, providing easier access to markets, customers for agricultural goods and wildlife products and employment, than the Northern study villages.

### 2.2 Survey methods

The survey took place from February 27 to March 19, 2021, in the 11 villages of the Northern and 7 villages of the Eastern sector of the Dja region. We surveyed 199 people, 99 in the Eastern sector villages and 100 in the Northern sector, representing 80% of households in the Northern sector and 56% of households in the Eastern sector (see Supplementary Materials S1). Respondents were chosen by their availability, their willingness to participate, and to ensure a balance of male and female respondents. All ethnic groups in the area were sampled as part of the study.

A semi‐structured questionnaire was developed for the study (see Supplementary Materials S2 for the full questionnaire). Researchers created a Kobo Collect form (www.kobtoolbox.org) to allow responses to be directly recorded on a tablet computer during the interview. The interview team consisted of two people; one person asked the questions and maintained the flow of conversation with the respondent, whilst the other recorded the responses. Interviews were also recorded using a telephone tape recorder with permission from the respondent.

Respondent ages ranged from 18 to 84 years with a mean age of 43 years, and the sample was slightly biased towards men (43% female and 57% male). None of the respondents had completed secondary education, 50% had attended secondary school at some point, 34% had attended but not completed primary school, and 16% had completed primary school. To explore household wealth, we used key indicators of wealth that were identified for villages in the same study area in 2019 (Brittain et al. in review). We first asked village chiefs to identify key indicators of relatively well‐off and poor households within their village, which were then checked in discussions with both men and women from the same villages. Each household was assigned to one of three categories: poor, medium, or well‐off, based on direct observations of these indicators (including the construction of the house and visible household assets). Amongst the household, 12% were in the ‘relatively well‐off’ category, 44% ‘average’ and 44% ‘poor’. There were no significant differences in respondents’ education level (Chi‐squared test; X^2^ = 1.77, df = 2, *p*‐value = 0.41) or wealth ranking (Chi‐squared test; X^2^ = 1.64, df = 2, *p*‐value = 0.44) between the two sectors.

Analyses were conducted in Microsoft Excel and R (R Core Team, 2020). We analysed results by sector rather than by village, noting the major differences between the sectors in access to markets and facilities.

### 2.3 Ethics

Ethical approval for the study was given through IIED’s Research Ethics Committee. The study was conducted using a Free Prior and Informed Consent (FPIC) approach. The research team first explained the aims of the research to the village chief and gained their permission to work and stay in the village. Once permission to work in the village was obtained, the team went from household to household to carry out the interviews. The team explained the objectives of the research and the types of questions that would be asked. It was explained that participation was entirely voluntary, that data would be anonymised, and the interviewee could opt out at any point. Free informed consent was verbally obtained by each respondent prior to their interview. No‐one under the age of 18 was selected for interview. Village locations were not recorded to ensure anonymity at the community level (St.John et al., 2016).

In the context of the current COVID‐19 pandemic, the interviews were carried out in compliance with barrier measures, which included wearing a mask (for investigators and respondents to whom masks were given) and keeping a distance of at least 1.50 m between investigators and respondents during interviews. In addition, the interviewers and respondents used hydro‐alcoholic hand washing gel before and after the interviews, and one soap was given to the respondents in addition to the masks, for washing their hands after the interviews.

## 3 RESULTS

### 3.1 Household livelihoods, hunting activity and wild meat consumption

**FIGURE 3:**
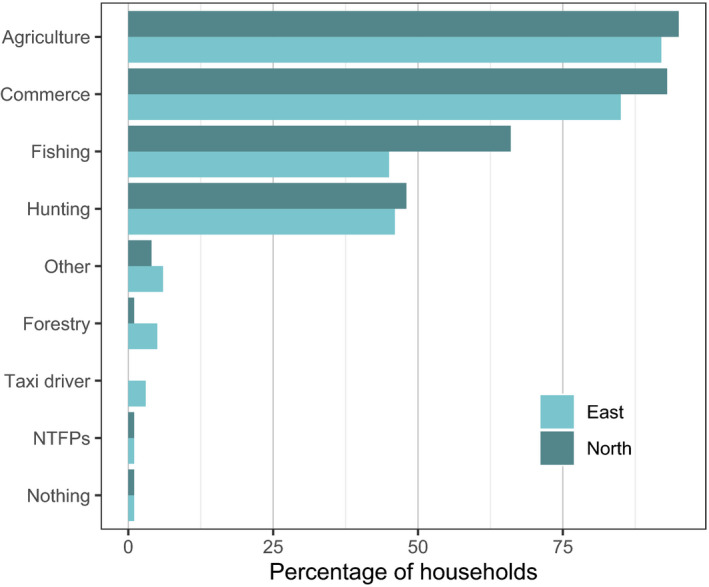
Main livelihood activities of respondents in the Eastern and Northern sector villages. *N* = 199 respondents

Household members in both sectors engaged in agriculture, hunting and fishing as their main livelihood activities (Figure 3), and in both sectors over 80% of households sold some of their produce. Very few households mentioned collection of Non‐Timber Forest Products (NTFPs), aside from wild meat, as a livelihood activity. This may be due to the seasonal nature of NTFP collection, with the main NTFP season (June – August) falling outside of our study period. Very few households had members with salaried employment. Several households had members employed in forestry, one respondent was a teacher and one worked in community radio. Others were self‐employed, including as moto taxi‐drivers, sawyers, tailors, hairdressers and house builders.

Just under 50% of households in both sectors reported members were involved in hunting as a livelihood activity. When asked more directly about whether anyone in their household hunted, a higher proportion (64% overall; 58% and 70% in the Eastern and Northern sector respectively) reported engaging in some hunting. Of these, 50% in the Eastern and 64% in the Northern sector engaged in trap hunting, 11% and 7% engaged in gun hunting, and a few households in the Northern sector also used spears (3%), machetes (2%) and arrows (1%) for hunting. There was no significant difference in the number of households reporting hunting between the Eastern and the Northern sector (Chi‐squared test; X^2^ = 2.04, df = 1, *p*‐value = 0.15).

The disparity between households reporting hunting as a livelihood activity and those reporting hunting activity when asked more directly, may reflect how households perceive hunting within the household. For example, some households may hunt with guns or set ‘barrier’ traps to prevent crop raiding, which may not be perceived as a livelihood activity by that household but rather a means to protect their main source of income.

**FIGURE 4:**
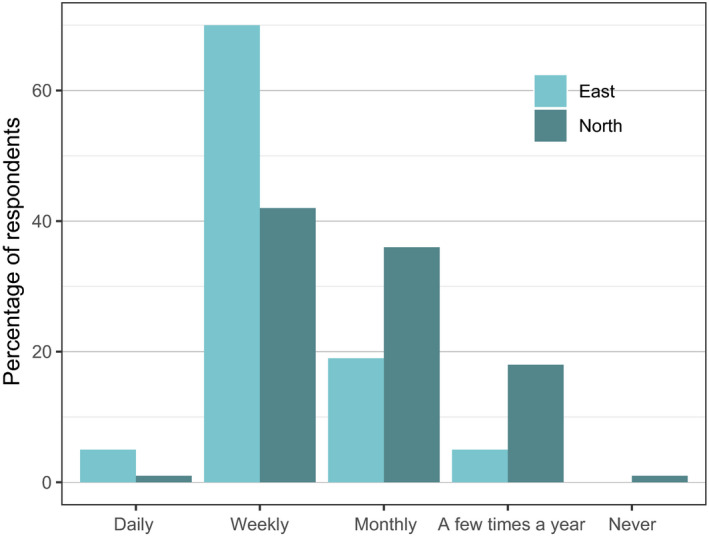
Frequency of wild meat consumption by respondents in Eastern and Northern sector villages. *N* = 199 respondents

Most respondents (60%) reported eating wild meat at least once a week (Figure 4). Reported frequency of wild meat consumption was significantly higher in the Northern sector (X‐squared = 22.9, df = 2, *p*‐value < 0.001).

### 3.2 Sources of information and levels of concern about COVID‐19

Many respondents were very concerned about the COVID‐19 pandemic (80% in the Eastern sector and 91% in the Northern sector; from multiple choice options of ‘not at all concerned’, ‘slightly concerned’ and ‘very concerned’). However, no respondent had knowingly caught COVID‐19 themselves, or knew anyone from their own village who had caught COVID‐19, and only 10% of respondents knew someone from outside the village who had caught COVID‐19.

**FIGURE 5:**
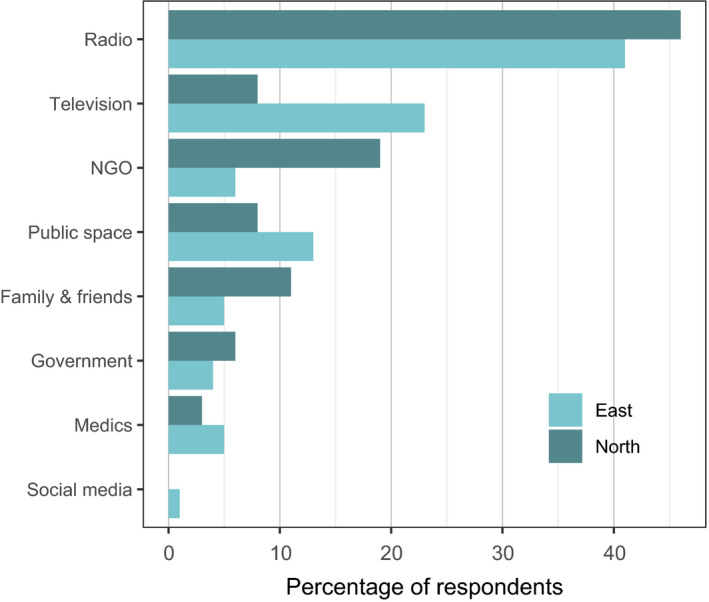
How respondents from Eastern and Northern sector villages first heard about COVID‐19. ‘Public space’ category includes village social spaces, schools and churches. ‘Government’ includes national, regional and local authorities. *N* = 199 respondents

Respondents were most likely to have first heard of COVID‐19 from the radio (Figure 5). In the Eastern sector, almost a quarter of respondents had heard of it from watching television, compared with only 8% of Northern sector respondents, probably due to lower levels of television ownership or access in the Northern sector where there was no mains electricity (CTKT, pers. obs). In comparison, 19% of Northern sector respondents received news of COVID‐19 from local conservation and development NGOs, compared with just 6% in the Eastern sector.

Most respondents thought that COVID‐19 had originated abroad, or been brought by foreigners, with 26% saying that it came from ‘white people’, 19% from the Chinese and 20% from foreign countries. Only 9% of respondents referenced wildlife, with 4.5% saying that COVID‐19 originated in a Cameroonian market, 2% saying that COVID‐19 came from pangolins, 1.5% from bats, 1% from small monkeys and 1% from great apes. A further 2% said that it came from ‘the air’, 1% from a Chinese laboratory and 23% said that they did not know.

Respondents were asked who they trusted to provide information on COVID‐19 from a pre‐defined list (see Supplementary Materials S2). High levels of trust were reported for information from government (80%), journalists (79%) and NGOs (78%) and lower for family and friends (55%) and meat sellers (38%). When asked to expand further on their choices, some respondents said that they did not trust meat sellers to provide good information, as they were motivated to make sales, and were not experts on wildlife. When asked for other sources that they trusted, respondents mentioned doctors and nurses (19% of respondents in the Northern sector and 1% in the Eastern), veterinarians (3% in the Eastern), hunters (1% in the Northern) and scientists (1% in the Northern).

### 3.3 Impacts of COVID‐19 on livelihoods, hunting activity and wild meat consumption

Most respondents (87%) reported having been ‘very much’ impacted by the response to COVID‐19, with 5% reporting being ‘a little’ impacted and 8% not at all impacted. There were no differences in these responses between the sectors (Chi‐squared test; X^2^ = 3.08, df = 2, *p*‐value = 0.21). The most frequently reported impacts of the government response to COVID‐19 were a loss of access to education, and travel restrictions, which led to loss of access to customers and loss of incomes (Table 1). Village schools and those in neighbouring towns were shut in response to the COVID‐19 pandemic from March to June 2020, resulting in children who would have stayed in town to go to school returning to the villages to be with their parents. Even whilst schools were closed, respondents reported that school fees were already paid. Meanwhile, transport costs increased (one example given was a doubling in the taxi fare to get to the capital city of Yaoundé from 5000 XAF (Central African CFA Franc) to 10000 XAF), and road maintenance and building projects in the region were halted, leaving roads that were difficult to use. The loss of customers due to travel restrictions resulted in reduced prices for crops (such as cocoa) and other products. One respondent reported that she previously sold palm wine in the village for 500 XAF and the price had now reduced to 350 XAF. Her clients were hunters, who now lacked customers due to the travel restrictions, and so did not have the money to buy her wine. Other respondents noted that they could no longer travel into town to sell fish, and one respondent noted that a buyer for their fish, who used to sell it in Equatorial Guinea, had stopped buying because the border between Cameroon and Equatorial Guinea had closed.

**TABLE 1:** The impacts of the government response to COVID‐19 on livelihoods in the Eastern and Northern sector villages

Activity/Asset	Eastern sector (99 respondents)		Northern sector (99 respondents)	
	Increase	Decrease	Increase	Decrease
Access to school	0	62	0	78
Income	0	38	1	52
Ability to travel	0	38	1	49
Access to customers	0	30	0	27
Access to work	0	17	0	9
Access to food	0	11	0	9
Access to goods	0	8	0	9
Access to markets	0	4	0	1
Food prices	0	2	0	1
Access to healthcare	0	2	0	0
Personal and/or family health	0	1	0	0

Note

Numbers represent the number of respondents who reported the impact. *N* = 198 respondents (99 in the eastern sector and 99 in the northern sector). The ‘increase’ and ‘decrease’ columns report how many respondents reported either increased or decreased access to a livelihood activity or household asset.

**FIGURE 6:**
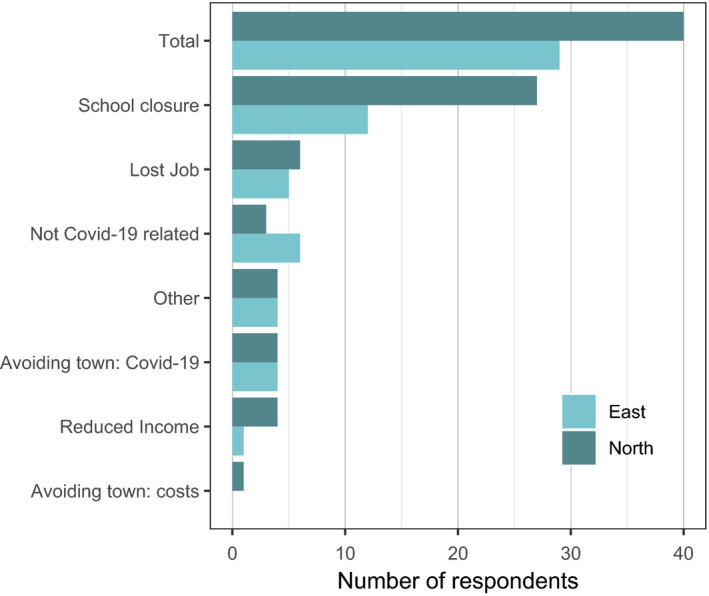
The number of respondents reporting a household member returning to the village during the pandemic, by the reason for their return. *N* = 199 respondents

Over half (53%) of the respondents had a household member who had returned to the village from living elsewhere, because of the COVID‐19 pandemic. In just under a quarter (23%) of these cases, the respondent themselves had returned to the village, but in most cases, children had returned (66%). Overall, more respondents in the Northern sector villages reported someone from their household returning to the villages during the pandemic. This difference was mainly due to a larger number of households having school children return in the Northern sector (Figure 6).

Many village children attend schools in nearby towns, living with relatives or friends during term time. Temporary school closures had resulted in many children returning to the village, increasing the number of dependents in each household. Other household members who had lost jobs outside of the village because of the pandemic had also returned. At the same time, transport restrictions and the cessation of road repair/construction work had restricted the availability of transport to and from town, increased transport costs and reduced the number of customers coming into the village to buy agricultural products and wild meat. Interviews suggested that these factors combined to reduce incomes, increase costs and reduce in food consumption for many local families. We did not attempt to quantify overall loss of incomes, but follow‐up interviews could be used to investigate this.

Respondents who had reported that the household did some hunting (64% of respondents) were asked whether their hunting activity had increased, decreased, or stayed the same during the pandemic. A quarter (25%) of these hunting households reported a decrease in their effort, compared to 73% who reported no change and 2% who reported an increase in effort. There were no significant differences between the two sectors (Chi‐squared test; X^2^ = 0.28, df = 2, *p*‐value = 0.87). Hunting activity was affected by economic shocks, reduced transport options and perceptions of disease risk. The number of clients travelling into the villages to buy wild meat declined, partly due to travel restrictions and increased travel costs, and partly, the respondents suggest, due to a reduced demand for wild meat, and this may in turn have reduced hunting effort.

When all 199 respondents were asked whether the pandemic had changed their eating habits, 32% of respondents reported changes. Significant differences were evident between sector; only 15% of respondents in the Eastern sector reported changes compared with 48% of respondents in the Northern sector (Chi‐squared = 23.8, df = 1, *p*‐value = <0.001). Of the 63 respondents that reported changes in eating habits, respondents most frequently reported a decrease in the amount of wild meat consumed (76%; 7 of 15 respondents in the Eastern sector, 41 of 48 respondents in the Northern sector), followed by a general reduction in all food consumption (22%; 8 and 6 respondents respectively), with one or two respondents in the Northern sector reporting changes in fish, poultry and red meat consumption (Supplementary Materials S3). Of the 48 respondents who had reduced their wild meat consumption, 89% (43) said that they had done so due to the risk of catching COVID‐19, whilst 8 said that it was because wild meat was less available, and 1 respondent said that it was because they were hunting less. Most of these respondents reported reducing consumption of pangolins (92%) great apes (31%) and small monkeys (29%; see Supplementary Materials S4).

### 3.4 Perceived disease risks from wild meat

Of the 43 respondents who reduced their consumption due to disease risk (22% of all respondents), all reported that they had stopped eating certain species because these species transmitted COVID‐19; of these, 38 specifically mentioned pangolins as carriers of COVID‐19. Eleven of these respondents said that they had been ‘told’ that pangolins (and less frequently monkeys, civets and great apes) could transmit COVID‐19. Who told them was not asked about by the interviewer, but one respondent mentioned that they had been told in December 2020, and another mentioned that they had been told during an NGO training session. One respondent said that the Government had told people not to eat pangolins due to the risk of COVID‐19 transmission. Several respondents suggested that hunters were preferentially not hunting pangolin, partly due to the worries about disease transmission and partly due to a reduction in demand.

**FIGURE 7:**
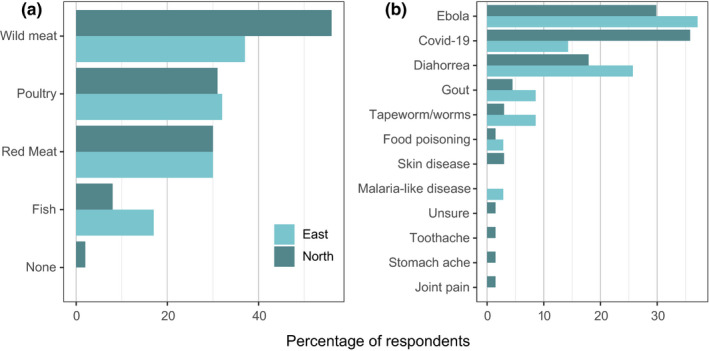
(a) The proportion of respondents in the Eastern and Northern sector who identified different types of meat as transmitting diseases. *N* = 199 respondents. (b) Reported diseases/ailments that can come from wild meat in the Eastern and Northern sector villages. *N* = 93 respondents. Results for other types of meat (poultry, red meat, fish) are provided in Supplementary Materials S5

All respondents were asked which types of meat could transmit diseases, given the options of wild meat, poultry, red meat, fish or none of the above. Wild meat was chosen by the most respondents (47%), although a high proportion of respondents also reported poultry and red meat as potentially transmitting diseases (32% and 30% respectively; Figure 7a). Only 2 respondents reported that none of these types of meat transmitted diseases. Respondents were then asked which diseases were transmitted by each type of meat that they had identified as bearing some disease risk. COVID‐19 was the most frequently disease reported as being transmitted by wild meat in the Northern sector villages, whilst Ebola was the most frequently mentioned in the Eastern sector villages (Figure 7b). Of the four given meat types, wild meat was the meat type most frequently reported to transmit COVID‐19, with 15% (29) of all respondents reporting that they thought COVID‐19 could be caught from wild meat, 6% from poultry, 4% from fish and 2% from red meat.

### 3.5 Reactions to wild meat market closures

Finally, we asked 193 respondents whether they agreed or disagreed with the idea of market closures with the following question: ‘*Some people have suggested wild animals should not be sold for food in urban markets to stop a future outbreak of a new disease*. *What do you think about the idea of closing urban wildlife markets*?’ Amongst the respondents, 73% disagreed, 19% agreed, and 8% neither agreed nor disagreed, with no significant difference in response seen between sectors (Chi‐squared test; X^2^ = 3.34, df = 2, *p*‐value = 0.19). When asked why they disagreed, as an open‐ended question, most respondents said that closing wild meat markets would significantly affect livelihoods, with 29% suggesting a ban would affect people's access to food and incomes, a further 27% mentioning reductions in income only and 20% mentioning a reduction in access to food only. With regard to the solutions to the closure of wild meat markets, 8% of respondents mentioned the need to set up alternative food and income sources to wild meat before instigating market closures, and 12% suggested that instead of market closures, markets should be regulated, or certain species banned from sale. Finally, 7% thought that markets should not be closed because they presented a low risk of disease transmission. Of the 37 respondents that agreed with the idea of a ban, 76% did so due to the potential disease risks. Two respondents suggested it was best to close markets as a precautionary measure, until more about the risks was known, one suggested partial closures for risky species, and one suggested markets should be closed due to unsustainable levels of hunting.

## 4 DISCUSSION

The COVID‐19 pandemic has resulted in significant economic shocks around the world and as hypothesised by McNamara et al. (2020), these have also been felt at the local level in Cameroon, affecting local livelihoods and wild meat use by communities surrounding the Dja reserve.

Cameroon has reported low rates of COVID‐19 cases compared with many world regions, although testing capacity is also low, and unsurprisingly, therefore, none of the respondents had knowingly contracted COVID‐19 or knew anyone locally who had suffered from it. However, a large proportion of respondents reported livelihood impacts of the pandemic, with almost 90% saying that they had been very much impacted. These impacts were predominantly due to nationally imposed lockdowns implemented before our survey, which had a range of ramifications for livelihoods and household budgets.

The picture on wild meat hunting and consumption was mixed. Although most hunters had not changed their effort, a sizeable minority had reduced it (25%), mostly due to the lack of ability to trade the meat to external clients. Interestingly, local consumption reductions appeared to be species‐specific. Of the 42 respondents who reduced their consumption of wild meat due to disease risk, all reported that they did so due to the perceived risk of COVID‐19, and 38 specifically mentioned reducing consumption of pangolins. We did not collect empirical data on hunting offtakes in the study villages, but a small but significant reduction in pangolin sales in Yaoundé has been reported by Harvey‐Carrol et al (in press), comparing market sales of pangolin in 2017 and 2018 with those of March – August 2020. This may suggest that hunting pressure on some of the threatened species in the area affected by wild meat consumption may have decreased; although how temporary this decrease was is hard to judge.

We did not ask what respondents were doing to replace income lost from agriculture and hunting; however, we were surprised to hear that on balance respondents were reducing their hunting activity rather than increasing it since it often acts as a safety net in times of need (the failure of crops or reduced incomes, such as in this case). This study asked people to gauge whether their consumption of different foods was increasing or decreasing, and as such is an extremely crude measure of impacts. Nevertheless, the finding that both overall food consumption and incomes are decreasing, in communities that are already impoverished, is cause for alarm.

The idea that COVID‐19 can be caught from wild meat may have come from discussions with NGOs, especially in the Northern sector where almost 10% of respondents originally heard about COVID‐19 from an NGO. Communities in the Northern sector decreased their consumption of wild meat significantly more than those in the Eastern sector, and this may be explained by the training sessions carried out by NGOs and the high degree of trust that the populations reported placing in these NGOs. It should also be noted that the populations in the Eastern sector of the Reserve benefit from better access to electricity and telephone reception, and therefore, have better access to information from other sources (Figure 5). These findings highlight the importance of clear and accurate communication from NGOs to local communities. Whilst conserving local wildlife populations is important for long‐term hunting sustainability, and wild meat consumption can pose significant health risks, communities should be given the best possible balanced information on disease risks. This is particularly the case in areas like the Dja where communities are reliant on wild meat for food and income and have few alternatives available. Based on scientific evidence it appears highly unlikely that COVID‐19 jumped from pangolins to humans (Frutos et al., 2020). NGOs should be aware of the potential ethical implications of talking about unproven links between wild meat consumption and COVID‐19, and about the consequences for their trusted status if these links are disproved in the future.

Many international NGOs have been vocal in their call for wild meat market bans in the wake of the COVID‐19 pandemic. The responses we obtained regarding the closure of markets reflect the importance of the sale and consumption of bushmeat for the communities around the Dja. Closing these markets would likely have significant consequences for the communities living around the Dja and for other communities who depend on bushmeat as a source of protein and income, especially when compounded with the other negative livelihood impacts of the pandemic that we have recorded here. However, respondents did express a range of views about wild meat market closures, suggesting that a more nuanced and risk‐based approach to wild meat policy could potentially be acceptable to local communities (as suggested by Booth et al., 2021).

Our study aimed to test the conceptual model created by McNamara et al. (2020) that tracks the likely implications for the wild meat trade of the systemic crisis triggered by COVID‐19 in sub‐Saharan Africa (Figure 1), in a real‐world situation. Our results generally support the causal pathways suggested by the model, including urban to rural migration due to unemployment and a reduction in demand for wild meat from consumers external to the village, which may be due to wider economic shocks. In the study villages, we identified factors that might lead to both increased wild meat use (increased number of dependents living at home, increased cost of buying food and goods from town, reduced local incomes) and decreased wild meat use (reduced demand from outside and within the village due to perceptions of disease risk; increased transport costs for clients and access to markets), with the net impact in this case being a reported decrease in wild meat consumption and hunting.

**FIGURE 8:**
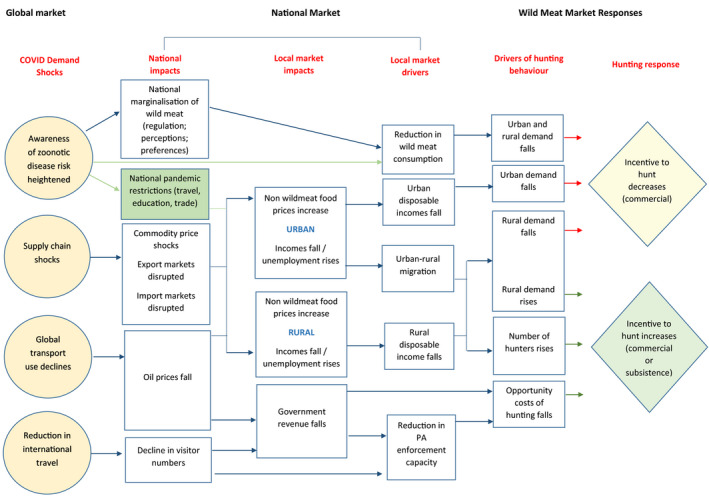
Revision of McNamara et al.’s (2020) causal model describing key linkages between global COVID‐linked shocks and wild meat market dynamics, including new pathways identified during this survey. Reproduced from McNamara et al., 2020

The study also highlights additional impact pathways not yet identified by McNamara et al. that could be included in the model. For example, the impact of national government responses to the pandemic, including road closures and transport restrictions on trade costs and school closures on household budgets, could arguably have affected household incomes around the Dja more than the trickle‐down effect of global economic shocks. In addition, the model focussed on changes in perception of disease risk from wild meat in urban areas as a driver of changes in wild meat demand. Although our study did not address urban demand (which may in principle have been partly responsible for reduced trade, along with travel restrictions), our study showed that perceptions of disease risk also changed consumption patterns in the study villages, at least for key species such as pangolin. We have incorporated these pathways into a revised model, presented in Figure 8.

In summary, the impacts of COVID‐19 on wild meat consumption in the Dja are likely to be species‐specific, with a reduction of the demand for pangolin and great ape meat during the study period due to concerns that these species transmit COVID‐19. This may or may not be a temporary impact, and further studies over the course of and after the pandemic would help provide a more long‐term analysis. However, the more concerning findings from this study are the loss of access to education and reductions in household incomes and overall food consumption. Whilst the importance of containing COVID‐19 is undisputed, even small reductions in household incomes during the pandemic could have longer‐term impacts on household livelihoods and food security, and the education of children in the community is a crucial tool in long‐term poverty reduction (Cotton, 2021). As the direct and indirect impacts of COVID‐19 look set to continue in Central Africa for the foreseeable future, communities should be supported by NGOs and local government to find ways of accessing local agricultural markets and selling their crops, and provision for schooling should be prioritised where possible, to ensure that the most vulnerable communities in are provided with a safety net in times of hardship.

## CONFLICT OF INTEREST

The co‐authors have no conflict of interest to declare.

## Supporting information

Supplementary Material

Supplementary Material

## Data Availability

The data that support the findings of this study are available from the corresponding author upon reasonable request.
