# Supplementary material for: Impacts of the COVID‐19 pandemic on livelihoods and wild meat use in communities surrounding the Dja Faunal Reserve, South‐East Cameroon
Source: Afr J Ecol. 2022 Mar 25;60(2):135–45. doi: 10.1111/aje.12995 (PMC9111389; doi:10.1111/aje.12995)
Supplement: Supplementary file 1 — Supplementary Material [file AJE-60-135-s002.docx]

Supplementary Material for: Impacts of the Covid-19 pandemic on livelihoods and wild meat use in communities surrounding the Dja Faunal Reserve, South-East Cameroon

Kamogne Tagne Cédric Thibaut, Steph Brittain, Dan Challender, Neil Maddison, EJ Milner-Gulland, Mama Mouamfon, Dilys Roe, Lauren Coad

**S1: Number of surveyed households in each of the surveyed villages in the Northern and Eastern sector**

| **Village ID** | **Number of interviews** | **Estimated total no. of households in village** | **Total % of households interviewed** |
| --- | --- | --- | --- |
| **North Antenna** |  |  |  |
| Village 1 | 8 | 8 | 100 |
| Village 2 | 7 | 9 | 78 |
| Village 3 | 12 | 15 | 80 |
| Village 4 | 8 | 10 | 80 |
| Village 5 | 3 | 5 | 60 |
| Village 6 | 8 | 11 | 73 |
| Village 7 | 14 | 15 | 93 |
| Village 8 | 14 | 18 | 78 |
| Village 9 | 7 | 7 | 100 |
| Village 10 | 9 | 12 | 75 |
| Village 11 | 10 | 15 | 66 |
| **East Antenna** |  |  |  |
| Village 12 | 10 | 15 | 66 |
| Village 13 | 16 | 40 | 40 |
| Village 14 | 10 | 20 | 50 |
| Village 15 | 15 | 25 | 60 |
| Village 16 | 15 | 18 | 83 |
| Village 17 | 18 | 25 | 72 |
| Village 18 | 15 | 35 | 43 |

**S2: Full Kobo Collect survey (see attached PDF)**

**S3: The number of respondents reporting changes in the consumption of different types of food, in each sector. Total number of respondents = 198 (99 in Eastern sector and 99 in Northern sector).**

| **Type of food** | **Direction of change** | **Number of respondents reporting change** | |
| --- | --- | --- | --- |
|  |  | **Eastern sector** | **Northern sector** |
| All food | decrease | 8 | 6 |
| Bushmeat | decrease | 7 | 41 |
| Red meat | decrease | 0 | 3 |
| Red meat | increase | 0 | 1 |
| Fish | decrease | 0 | 1 |
| Fish | increase | 0 | 1 |
| Poultry | decrease | 0 | 1 |
| Poultry | increase | 0 | 1 |
| Total respondents reporting a change in food consumption | | 15 | 48 |

**S4: Number of respondents (of the 48 who reported changes in wild meat consumption; 41 in the northern sector and 7 in the eastern sector) reporting reductions in the consumption of specific species/taxa.**

|  | **Number of respondents** | | |
| --- | --- | --- | --- |
| **Species/taxa** | **Northern sector (of 41)** | **Eastern Sector (of 7)** | **Total number (of 48) and percentage** |
| Pangolin sp. | 39 | 5 | 44 (92%) |
| Great apes | 14 | 1 | 15 (31%) |
| Small monkeys | 13 | 1 | 14 (29%) |
| Genets and civets | 3 | 0 | 3 (6%) |
| Red duiker | 1 | 0 | 1 (2%) |
| Blue duiker | 1 | 0 | 1 (2%) |
| Yellow backed duiker | 1 | 0 | 1 (2%) |
| Bush pig | 1 | 0 | 1 (2%) |
| Rat sp. | 1 | 0 | 1 (2%) |
| Bat | 1 | 0 | 1 (2%) |
| Cat | 1 | 0 | 1 (2%) |
| Dog | 1 | 0 | 1 (2%) |

**S5: Diseases/ailments reported for different types of meat/fish, for the 130 respondents (59 in the eastern sector and 71 in the northern sector) that reported meat/fish as transmitting disease. The total percentage describes the percentage of respondents reporting each ailment, of the 130 that reported that meat/fish could transmit disease.**

| **Type of meat/fish** | **Disease/ailment** | **Number of respondents reporting disease/ailment** | | |
| --- | --- | --- | --- | --- |
|  |  | **Eastern sector (of 59)** | **Northern sector (of 71)** | **Total number (of 130) (and percentage to nearest 1%)** |
| Poultry | Beri Beri | 1 | 0 | 1 (1%) |
| Poultry | Bird flu | 21 | 28 | 49 (38%) |
| Fish | Bird flu | 0 | 1 | 1 (1%) |
| Red Meat | Cancer | 0 | 1 | 1 (1%) |
| Fish | Chemical poisoning | 1 | 0 | 1 (1%) |
| Fish | Constipation | 0 | 2 | 2 (2%) |
| Red Meat | Covid-19 | 0 | 3 | 3 (2%) |
| Poultry | Covid-19 | 2 | 9 | 11 (8%) |
| Wild Meat | Covid-19 | 5 | 24 | 29 (22%) |
| Fish | Covid-19 | 5 | 2 | 7 (5%) |
| Red Meat | Diarrhoea | 5 | 3 | 8 (6%) |
| Poultry | Diarrhoea | 1 | 0 | 1 (1%) |
| Wild Meat | Diarrhoea | 9 | 12 | 21 (16%) |
| Fish | Diarrhoea | 1 | 0 | 1 (1%) |
| Poultry | Ebola | 4 | 13 | 17 (13%) |
| Wild Meat | Ebola | 13 | 20 | 33 (25%) |
| Fish | Ebola | 5 | 2 | 7 (5%) |
| Red Meat | Epilepsy | 0 | 2 | 2 (2%) |
| Red Meat | Food poisoning | 0 | 2 | 2 (2%) |
| Poultry | Food poisoning | 0 | 1 | 1 (1%) |
| Wild Meat | Food poisoning | 1 | 1 | 2 (2%) |
| Fish | Food poisoning | 5 | 2 | 7 (5%) |
| Poultry | Gonococcus | 1 | 0 | 1 (1%) |
| Red Meat | Gout | 1 | 0 | 1 (1%) |
| Wild Meat | Gout | 3 | 3 | 6 (5%) |
| Wild Meat | Joint pain | 0 | 1 | 1 (1%) |
| Red Meat | Leprosy | 1 | 0 | 1 (1%) |
| Fish | Malaria | 5 | 2 | 7 (5%) |
| Red Meat | Malaria-like disease | 1 | 0 | 1 (1%) |
| Poultry | Malaria-like disease | 1 | 0 | 1 (1%) |
| Wild Meat | Malaria-like disease | 1 | 0 | 1 (1%) |
| Fish | Malaria-like disease | 1 | 0 | 1 (1%) |
| Red Meat | Parasites | 0 | 2 | 2 (2%) |
| Red Meat | Pig flu | 1 | 0 | 1 (1%) |
| Red Meat | Plague | 3 | 2 | 5 (4%) |
| Fish | Polluted water | 2 | 0 | 2 (2%) |
| Poultry | Skin disease | 0 | 1 | 1 (1%) |
| Wild Meat | Skin disease | 0 | 2 | 2 (2%) |
| Wild Meat | Stomach-ache | 0 | 1 | 1 (1%) |
| Red Meat | Tapeworm | 20 | 11 | 31 (24%) |
| Wild Meat | Tapeworm/worms | 3 | 2 | 5 (4%) |
| Wild Meat | Toothache | 0 | 1 | 1 (1%) |
| Red Meat | Unknown disease | 2 | 0 | 2 (2%) |
| Fish | Unknown disease | 10 | 0 | 10 (8%) |
| Red Meat | Unsure | 0 | 2 | 2 (2%) |
| Poultry | Unsure | 1 | 1 | 2 (2%) |
| Wild Meat | Unsure | 0 | 1 | 1 (1%) |
| Fish | Unsure | 1 | 1 | 2 (2%) |
| Poultry | Worms | 1 | 0 | 1 (1%) |
