# Supplementary material for: Impacts of the COVID‐19 pandemic on livelihoods and wild meat use in communities surrounding the Dja Faunal Reserve, South‐East Cameroon
Source: Afr J Ecol. 2022 Mar 25;60(2):135–45. doi: 10.1111/aje.12995 (PMC9111389; doi:10.1111/aje.12995)
Supplement: Supplementary file 2 — Supplementary Material [file AJE-60-135-s001.pdf]

Cameroon Bushmeat Covid Survey

Name

____

Age

____

Gender

- ◯ Male
- ◯ Female

Primary Livelihood Activities

Ask them the question and then choose yourself from the options, or choose 'other' and add details if their answer is not represented in any of these options

- ☐ Hunting
- ☐ Agriculture
- ☐ Commerce
- ☐ Fishing
- ☐ Forestry
- ☐ Retired
- ☐ Nothing
- ☐ Other (write in below)

Give details of livelihood activity:

____

Village name

- ◯ Village name 1
- ◯ Village name 2
- ◯ Village name 3
- ◯ Village name 4
- ◯ Village name 5
- ◯ Village name 6
- ◯ Village name 7
- ◯ Village name 8
- ◯ Village name 9
- ◯ Village name 10

When did you first hear about Covid-19?

Either estimate the month, or give the number of weeks ago.

How did you hear about it?

Ask them the question and then choose yourself from the options, or choose 'other' and add details if their answer is not represented in any of these options

- [ ] Radio
- [ ] Newspaper (online or paper)
- [ ] TV
- [ ] Family
- [ ] Friends
- [ ] Public space (bar/shop)
- [ ] Social media - whatsapp
- [ ] Social media - facebook
- [ ] Other (give details)

Give details of how they heard about it:

Where did Covid-19 come from?

Ask them the question and then choose yourself from the options, or choose 'other' and add details if their answer is not represented in any of these options

- [ ] Chinese laboratory
- [ ] Chinese bushmeat market
- [ ] Cameroon bushmeat market
- [ ] From the Chinese
- [ ] from white people
- [ ] species: pangolin
- [ ] species: small monkeys
- [ ] species: bats
- [ ] species: great apes
- [ ] bushmeat (general)
- [ ] don't know
- [ ] Other

Give details of where they think disease comes from:

How concerned are you about Covid-19?

Ask them to choose from the options below

- ◯ Very concerned
- ◯ Slightly concerned
- ◯ Not at all concerned

Why do you have this level of concern/are you not concerned?

____

Are you taking any steps to protect yourself and your household against Covid-19?

- ◯ Yes
- ◯ No
- ◯ I don't know how to

What measures have you take over the last year to protect yourself and the household against Covid-19?

Ask them the question and then choose yourself from the options, or choose 'other' and add details if their answer is not represented in any of these options

- ☐ Washing hands
- ☐ Wearing masks
- ☐ Reducing travel
- ☐ Social distancing (keeping distant from other people)
- ☐ Changes in what the household eats (specify)
- ☐ Changing livelihood activities (specify)
- ☐ Other

Give details of the other measures they are taking:

____

Have you or anyone you know had Covid-19? If yes, who?

Ask them the question and then choose yourself from the options, or choose 'other' and add details if their answer is not represented in any of these options

- ☐ No
- ☐ Myself
- ☐ Someone in the household
- ☐ Someone in the village
- ☐ Someone in another village/town in the same district
- ☐ Someone in another village/town further away
- ☐ A stranger visiting the village
- ☐ Other

Give details of the person that they know that caught covid, if 'other' was selected

____

Has the government response to Covid-19 affected you and your family at all?

Ask them to choose from the options below

- ◯ Yes, very much
- ◯ Yes, a little
- ◯ No
- ◯ Don't know

**If yes, how has your life been affected?**<br>*Ask them the question, rather than providing them with the options. But the options can be used as prompts*	Increased	Decreased
**Ability to travel**	◯	◯
**Access to healthcare**	◯	◯
**Access to buying goods (petrol, oil, soap etc)**	◯	◯
**Access to food**	◯	◯
**Food prices**	◯	◯
**Access to work**	◯	◯
**Income**	◯	◯
**Personal/family health**	◯	◯
**Access to markets for selling goods**	◯	◯
**Access to customers**	◯	◯
**Access to school**	◯	◯

If the intervewee suggested that their life has been affected, write details of how and why here:

____

Were you and everyone else in your household based in the village before the beginning of the pandemic/the past year or were any of you working/living elsewhere?

This is to find out whether any family members who were living outside of the village have since returned to the village for reasons connected to the covid-19 pandemic

- ◯ Based in the village
- ◯ Living elsewhere

Which family members were away?

- [ ] Myself
- [ ] Wife/partner
- [ ] Husband/partner
- [ ] Entire family
- [ ] Mother
- [ ] Father
- [ ] Sister(s)
- [ ] Brother(s)
- [ ] Child(ren))
- [ ] Extended family (Aunts, cousins, etc)
- [ ] Grandparent

Why did you/family members come back to the village?

Ask the question, and then select whether the answer was covid-19 relevant. Write any notes on the reason in the following text box

- ( ) Covid-19
- ( ) Not Covid-19

Write any notes here about why the interviewee or their family members came back to the village

____

When did you/they return? (estimate days/weeks/months ago)

____

What are you/they doing now in the village for money?

Ask them the question and then choose yourself from the options, or choose 'other' and add details if their answer is not represented in any of these options

- [ ] Agriculture
- [ ] Hunting
- [ ] Fishing
- [ ] Commerce
- [ ] Forestry
- [ ] Retired
- [ ] Nothing
- [ ] Other (write below)

Give details of the other activities they are doing for money here:

____

Which of these choices is closest to how often you normally consume bushmeat?

Give them these options to choose from

- ○ Daily
- ○ Weekly
- ○ Monthly
- ○ A few times a year
- ○ Never

Have your eating habits changed during the Covid-19 pandemic?

(make sure that if people say yes, their reasons are actually the pandemic)

- ○ Yes
- ○ No

If you answered yes, how have you changed how much food, and different meats you eat?

Ask them for each of the food categories

	increase	decrease	same
**all food**	○	○	○
**bushmeat**	○	○	○
**red domestic meat (e.g. pigs, goat, sheep, beef)**	○	○	○
**fish**	○	○	○
**poultry**	○	○	○

Give details on how and why their eating habits have changed here:

This can include other foods that have changed, and why any changes have taken place for any of the foods

____

Why has Covid-19 affected how often you eat bushmeat?

If they specify that they have changed the amount of bushmeat they are eating, ask this question. Ask them the question and then choose yourself from the options, or choose 'other' and add details if their answer is not represented in any of these options

- ☐ Risk of disease
- ☐ Bushmeat is less available
- ☐ Bushmeat is more available
- ☐ Alternatives (e.g. domestic meat, fish) less available
- ☐ Other (give reason)

Give details on the other reasons Covid-19 has changed how often they eat bushmeat here:

____

Are there certain bushmeat species you are either eating more or less of during the Covid-19 pandemic? Which ones?

Ask them the question and then choose yourself from the options, or choose 'other' and add details if their answer is not represented in any of these options. You can use the options as a prompt

	More	Less
pangolin	○	○
porcupine	○	○
cane rat	○	○
small monkeys	○	○
bush pigs	○	○
red duiker	○	○
great apes	○	○
blue duiker	○	○
antelope	○	○
genets and civets	○	○
hyrax	○	○
snakes	○	○
crocodiles	○	○
monitor lizard	○	○
other	○	○

Why has Covid-19 meant that you are eating more/less of those species?

(only ask this if they have reported changes to the amount they eat in the question above)

Has where you get your food from changed during the Covid-19 pandemic?

(make sure that if people say yes, their reasons are actually the pandemic, and not just seasonal changes)

	Eating more	Eating less	no change
**Hunted/trapped bushmeat**	○	○	○
**Bought bushmeat**	○	○	○
**Own produced red domestic meat (e.g. pig, goat, sheep, cow)**	○	○	○
**Bought red domestic meat (e.g. pig, goat, sheep, cow)**	○	○	○
**Fished fish**	○	○	○

**Bought fish**	○	○	○
**Own produced poultry**	○	○	○
**Bought poultry**	○	○	○
**Own-produced food in general**	○	○	○
**Bought food in general**	○	○	○

Provide any notes on changes to where they get their food from here:

____

Do you hunt/trap for food?

- [ ] No
- [ ] Gun Hunt
- [ ] Trap Hunt
- [ ] Other hunting type (give hunting type)

Give details of the other hunting type(s) here

____

**Has the amount of time you spend hunting/trapping changed during the Covid-19 pandemic, and why?**	Same amount	Increased	Decreased
**All hunting**	○	○	○
**Trap hunting**	○	○	○
**Gun hunting**	○	○	○
**Other hunting**	○	○	○

Why do you spend more/less time hunting than before the Covid-19 pandemic?
(only ask this question if they have said that they have changed their hunting activity during the pandemic)

____

Which of the following foods do you think can give you diseases?
give them the options below. If they choose bushmeat then more questions on bushmeat will appear in the survey

- [ ] Red meat, such as pork, beef, sheep and goat
- [ ] Poultry
- [ ] Bushmeat
- [ ] Fish
- [ ] All have the same risks
- [ ] None have risks

What diseases do you think that red meat spreads?

Ask them the question and then choose yourself from the options, or choose 'other' and add details if their answer is not represented in any of these options.

- [ ] Salmonella/ food poisoning
- [ ] Ameobic Dysentry/ diahorrea
- [ ] Malaria
- [ ] Ebola
- [ ] Covid-19
- [ ] Other
- [ ] Unsure

Give details of other diseases red meat spreads here:

If they are specifying other diseases, make sure you say which type of meat spreads the disease

---

What diseases do you think that poultry spreads?

Ask them the question and then choose yourself from the options, or choose 'other' and add details if their answer is not represented in any of these options

- [ ] Salmonella/ food poisoning
- [ ] Ameobic Dysentry/ diahorrea
- [ ] Malaria
- [ ] Ebola
- [ ] Covid-19
- [ ] Other
- [ ] Unsure

Give details of other diseases poultry spreads here:

If they are specifying other diseases, make sure you say which type of meat spreads the disease

---

What diseases do you think that bushmeat spreads?

Ask them the question and then choose yourself from the options, or choose 'other' and add details if their answer is not represented in any of these options

- [ ] Salmonella/ food poisoning
- [ ] Ameobic Dysentry/ diahorrea
- [ ] Malaria
- [ ] Ebola
- [ ] Covid-19
- [ ] Other
- [ ] Unsure

Give details of other diseases bushmeat spreads here:

If they are specifying other diseases, make sure you say which type of meat spreads the disease

What diseases do you think that fish spreads?

Ask them the question and then choose yourself from the options, or choose 'other' and add details if their answer is not represented in any of these options

- [ ] Salmonella/ food poisoning
- [ ] Ameobic Dysentry/ diahorrea
- [ ] Malaria
- [ ] Ebola
- [ ] Covid-19
- [ ] Other
- [ ] Unsure

Give details of other diseases fish spread here:

If they are specifying other diseases, make sure you say which type of meat spreads the disease

How do you think disease is spread by red meat?

Ask them the question and then choose yourself from the options, or choose 'other' and add details if their answer is not represented in any of these options

- [ ] By eating it
- [ ] By touching it
- [ ] Mixed blood
- [ ] Taboos (e.g. by killing certain species, you get sick)
- [ ] Other

Give details of other ways that red meat spreads disease here:

How do you think disease is spread by poultry?

Ask them the question and then choose yourself from the options, or choose 'other' and add details if their answer is not represented in any of these options

- [ ] By eating it
- [ ] By touching it
- [ ] Mixed blood
- [ ] Taboos (e.g. by killing certain species, you get sick)
- [ ] Other

Give details of other ways that poultry spreads disease here:

How do you think disease is spread by bushmeat?

Ask them the question and then choose yourself from the options, or choose 'other' and add details if their answer is not represented in any of these options

- [ ] By eating it
- [ ] By touching it
- [ ] Mixed blood
- [ ] Taboos (e.g. by killing certain species, you get sick)
- [ ] Other

Give details of other ways that bushmeat spreads disease here:

____

How do you think disease is spread by fish?

Ask them the question and then choose yourself from the options, or choose 'other' and add details if their answer is not represented in any of these options

- [ ] By eating it
- [ ] By touching it
- [ ] Mixed blood
- [ ] Taboos (e.g. by killing certain species, you get sick)
- [ ] Other

Give details of other ways that fish spreads disease here:

____

Who do you trust the most to provide reliable information about the risk of getting disease from meat?

you can provide them with the options below and ask them for any others

- [ ] Friends and Family
- [ ] Government
- [ ] NGOs
- [ ] Journalists
- [ ] Meat sellers
- [ ] No one
- [ ] Other

Give details of who they trust and why:

____

Some people have suggested wild animals should not be sold for food in urban markets to stop a future outbreak of a new disease. What do you think about the idea of closing urban wildlife markets?

- ◯ Agree
- ◯ Disagree
- ◯ Neither agree nor dissagree

Why do you think this?

____

What is the highest level of education you have received?

- ◯ Not completed Primary
- ◯ Completed Primary
- ◯ Attended Secondary
- ◯ Gained the BAC
- ◯ Attended university/higher eduation
- ◯ Prefer not to say
- ◯ None

Researcher estimation of the wealth category of the household, based on visual estimation of the house

Look at what the house and roof is made of, and make an estimate on whether this is a very rich or very poor house, or an 'average' house.

- ◯ Rich
- ◯ Average
- ◯ Poor
